# 

**Published:** 1895-09-21

**Authors:** 


					<aB~ "^"^ABSOLUTEL^^URE, therefore BEST. Septkmbkb 21st. mt
OADBURY'S ^ CADBURYS
COCOA
tH|
COCOA
' Of absolute ntirW AC A c ii is ?? igL r^r- i "The Typical Cocoa of English Manufacture-
aosolute purity,and freedom fromjilkah.^^ Absolutely Pure."- the Analyst.
s^hupm s?E?e5?=i-^i2vi9s^
-~5^Ci.ftscoir Western Infirmary
WiCH HOSPI TAL
. _    ?=n w -- ??? .???nil ruFUT PRICE TWOPENCE.
No. 469^Voi xyiii WITH EXTRA NUR8ING 8UPPLEMENT. aas^frSMSC
s,Jt 21sM895.

				

